# How is COVID-19 altering the manufacturing landscape? A literature review of imminent challenges and management interventions

**DOI:** 10.1007/s10479-021-04397-2

**Published:** 2021-11-17

**Authors:** Kawaljeet Kapoor, Ali Ziaee Bigdeli, Yogesh K. Dwivedi, Ramakrishnan Raman

**Affiliations:** 1 Aston University, Birmingham, UK; 2 Emerging Markets Research Centre (EMaRC), School of Management, Swansea University, Room #323, Bay Campus, Fabian Bay, Swansea, SA1 8EN Wales, UK; 3 Symbiosis Institute of Business Management, Pune & Symbiosis International (Deemed University), Pune, India

**Keywords:** COVID-19 challenges, Digital technologies, Manufacturing, Pandemic, Repurposing

## Abstract

Disruption from the COVID-19 pandemic has caused major upheavals for manufacturing, and has severe implications for production networks, and the demand and supply chains underpinning manufacturing operations. This paper is the first of its kind to pull together research on both—the pandemic-related challenges and the management interventions in a manufacturing context. This systematic literature review reveals the frailty of supply chains and production networks in withstanding the pressures of lockdowns and other safety protocols, including product and workforce shortages. These, altogether, have led to closed facilities, reduced capacities, increased costs, and severe economic uncertainty for manufacturing businesses. In managing these challenges and stabilising their operations, manufacturers are urgently intervening by—investing in digital technologies, undertaking resource redistribution and repurposing, regionalizing and localizing, servitizing, and targeting policies that can help them survive in this altered economy. Based on holistic analysis of these challenges and interventions, this review proposes an extensive research agenda for future studies to pursue.

## Introduction

This is not the first time that manufacturing has faced large-scale disruptions; natural disasters, trade wars, political risks, and other infectious diseases have previously put the industry in jeopardy (Okorie et al., 2020). Still, COVID-19 precedes all, as it adversely challenges the global economy (Handfield et al., 2020). For instance—at one point during the pandemic, US industrial production recorded the largest monthly decline since the Second World War (Rapaccini et al., 2020); the UK manufacturing system is still experiencing massive economic shocks after years of impending Brexit and deindustrialisation (Harris et al., 2020). Even before the pandemic, manufacturing production in 2019 recorded a global economic slowdown, which has now turned into a global economic crisis with COVID-19 (Teng et al., 2021). Despite past disasters, lessons have not been learnt sufficiently for manufacturing to successfully tackle the challenges of the pandemic (Handfield et al., 2020; Javaid et al., 2020a).

Manufacturers continually battle challenges of liquidity and profitability, and now COVID-19 is making them even more vulnerable to economic shocks (Juergensen et al., 2020). Now, situated in the middle of an economic storm, manufacturing is facing difficulties with cancelled orders, poor revenues, and falling stock prices (Handfield et al., 2020; Tian et al., 2021; Wuest et al., 2020). Such instabilities and unpredictable market environments (Linton & Vakil, 2020; Paul & Chowdhury, 2020b) are creating panic in the industry, resulting in market anomalies and distorted supply–demand patterns (Khoo & Hock, 2020). A clear understanding of the new challenges is necessary to be able to devise suitable interventions (strategies and solutions) that can ensure the sustainability of manufacturing businesses. Therefore, using recent literature, this review will collate the challenges currently faced by manufacturers and discuss interventions that could potentially minimize or manage these challenges. It is certain that the pandemic will have a permanent effect on how firms navigate their strategic choices, and how governments regulate polices around manufacturing and global trade in the future (Dür et al., 2020; Pinna & Lodi, 2021). There are likely to be modifications to supply chains to incorporate more flexible processes, and innovative product planning will take into account future uncertainties and/or global emergencies (Sakhardande & Gaonkar, 2021). It is important to document such interventions to help prepare the manufacturing industry for future pandemic-level events.

The significance of this review is two-fold. Firstly, manufacturing takes a leading role in maintaining stability across world economies (Luo et al., 2020), which makes it a prime and timely topic warranting significant research attention in COVID-times. To the best of our knowledge, this review is the first of its kind to pull together recent research on COVID-19 challenges and management interventions in a manufacturing context. While reviews, such as Queiroz et al. (2020) are available, their focus is on the impact of epidemic outbreaks, in general, on supply chains, in particular. This review, however, targets the challenges of COVID-19, alongside corresponding interventions that are not limited to supply chain challenges but are more inclusive of overarching challenges hindering the survival of manufacturing businesses. Secondly, significant research is available on COVID-19 (Choi, 2021; Gupta et al., 2021; Khalilpourazari & Doulabi, 2021) in a supply chain context (Ivanov & Dolgui, 2020; Ivanov, 2021a), humanitarian logistics in an epidemic context (Kumar et al., 2021; Wamba, 2020), and in other contexts (Adamovic, 2022; Chipidza, 2021; Dwivedi et al., 2020; Islam et al., 2022; Papadopoulos et al., 2020; Shirish et al., 2021; Trkman et al., 2021). However, an accumulative understanding of such a widespread event in manufacturing is rather limited (Hilmola et al., 2020; Paul & Chowdhury, 2020b; Queiroz et al., 2020). This gap in the literature further justifies the importance of our review.

With COVID-19, more researchers are exploring the diverse challenges causing production disruptions, whilst also looking at potential recovery strategies (Belhadi et al., 2021). Therefore, this study aims to identify, interpret, and summarize the literature available on both the pandemic-related challenges and proposed interventions for manufacturing. To guide this review, we ask the following research questions (RQs)—RQ1: How has the pandemic disrupted the manufacturing landscape to create new challenges? RQ2: What guidance is there in the literature for effectively managing these challenges? RQ3: On which overarching research themes should future research focus, for tackling pandemic-related challenges in manufacturing?

In terms of contributions, firstly, this study categorizes the research investigating the relationship between COVID-19 and manufacturing. Secondly, in debating the extremity and efficacy of pandemic-related challenges and their interventions, respectively, we propose a research agenda that identifies potential avenues for furthering research on the topic. Thirdly, the findings enhance our understanding of how ready manufacturers now are to be able to respond to global emergencies. This will help inform practitioners’ and policymakers’ decisions in safeguarding the manufacturing industry from future disruptions.

The rest of the paper is structured as follows—we explain the systematic literature review followed in this paper in Sect. 2, and then move on to discuss the findings of this review in Sect. 3. Next, the challenges (Sect. 3.2) and management interventions (Sect. 3.3) are reviewed, which are reasoned and further discussed in Sect. 4, alongside the limitations and implications of this review. The study is drawn to a close with conclusions in Sect. 5.

## Methodology

**Fig. 1:**
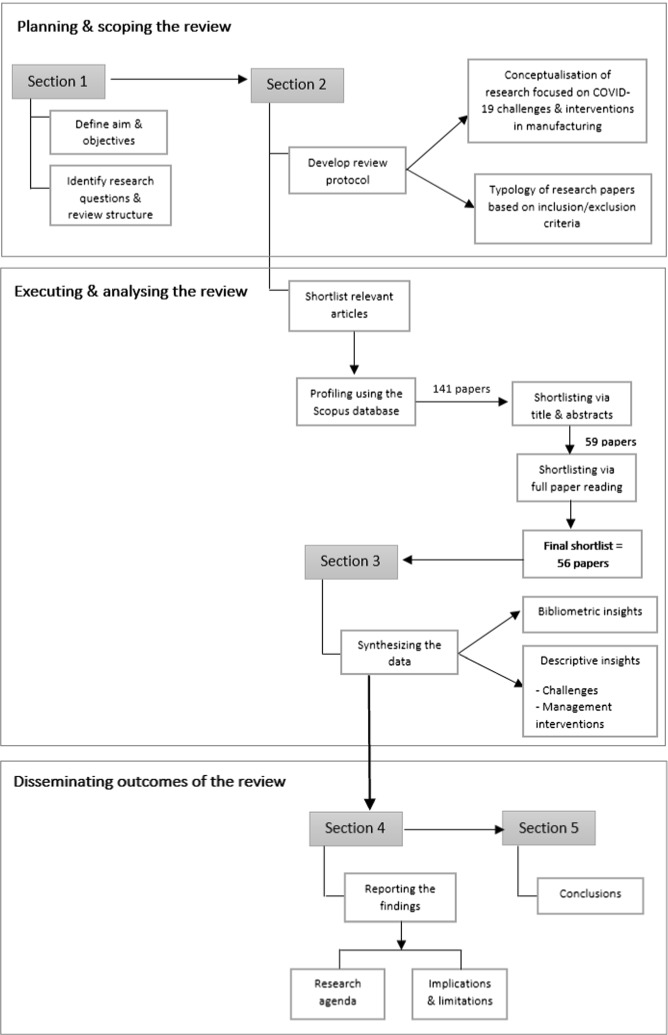
Systematic literature review

The review adopted a systematic literature review (SLR) method (Tranfield et al., 2003). This allows a transparent elicitation of key issues for the topic of interest, which can then be analysed using a standard protocol (Boell & Cecez-Kecmanovic, 2015). Existing research in the manufacturing and supply chain contexts has successfully employed the SLR approach to generate valuable insights (Kamal et al., 2020; Kapoor, et al., 2021a, 2021b; Pereira et al., 2014; Queiroz et al., 2020). A three-stage approach was used (Delbufalo, 2012; Tranfield et al., 2003): (a) *planning and scoping the review process—*here, the aim and objectives for this review were identified and a review protocol was established; (b) *executing and analysing the review—*here, the focus was on shortlisting relevant articles and synthesizing those articles to summarize meaningful evaluations; and (c) *disseminating the outcomes of the review—*here, systematic reporting of the findings was undertaken (Fig. 1).

### Research protocol

The research protocol, following the approach suggested by Tranfield et al. (2003), was focused on *how COVID-19 has impacted business operations across manufacturing firms.* The scope of the research domain included both qualitative and quantitative empirical papers on the topic. The research protocol elucidated details on: (a) manufacturing challenges due to COVID-19 and (b) interventions made to manage COVID-19 challenges in manufacturing. Numerous conditions were listed to justify the typology of research papers to be included in this review—the inclusion/exclusion criteria are identified herein:

- We used the largest database of peer-reviewed articles—Elsevier’s Scopus database—to search for publications that apply to this review.
- Given the limited amount of research on the topic, the search included articles published in both peer-reviewed journals and conference proceedings.
- Restrictions were applied to include articles only published after March 2020, as WHO declared COVID-19 as a pandemic in the March of 2020 (WHO, 2020). Additionally, only articles published in the English language were shortlisted.
- The substantive relevance of articles was confirmed by employing a keyword search in extracting relevant articles from the database. The keyword selection criteria were based on the research questions and ultimately focused on terms relating to both the COVID-19 pandemic and manufacturing. We used different terms for the *pandemic*, including ‘*Coronavirus*’ (with and without a space), ‘*COVID-19*’ (with and without a hyphen), and these were combined with words such as *challenges* and *threats* to generate relevant research in the manufacturing context.
- Specific keyword combinations were ‘*manufacturing and pandemic*’, or ‘*manufacturing and COVID* (or *COVID-19* or *COVID19* or *COVID 19* or *Coronavirus* or *Corona Virus*)’, or ‘*manufacturing challenges and COVID* (or *COVID-19* or *COVID19* or *COVID 19* or *Coronavirus* or *Corona Virus*)’, or ‘*manufacturing and challenges and COVID* (or *COVID-19* or *COVID19* or *COVID 19* or *Coronavirus* or *Corona Virus*)’, or ‘*manufacturing challenges and pandemic*’, or ‘*manufacturing and challenges and pandemic*’, or ‘*manufacturing and threats and pandemic*’, or ‘*manufacturing and threats and COVID* (or *COVID-19* or *COVID19* or *COVID 19* or *Coronavirus* or *Corona Virus*)’.
- There were no restrictions applied to the methodology followed in the shortlisted papers. Irrespective of their empirical and conceptual nature, an all-inclusive shortlist was created to include case studies, survey-based articles, and analytical papers reporting insights on COVID-19 challenges for manufacturers, and the types of management strategies applied to tackle such challenges.

### Results of the database search

The search string identified above returned 141 articles altogether. For analysis, a review of the 141 publications by *title* and *keywords* was carried out to include only articles with a clear focus on manufacturing in a COVID-19 context. Reviewing these abstracts reduced the shortlist to 59 articles, which were then read in full to confirm their applicability and extract relevant insights aligned with the research objectives of this review. Reading the full articles revealed that not all the studies fitted the scope of this review; for instance, Liu et al., (2021a, 2021b) have sharing economy as the main focus of their study; although they briefly discuss a COVID-scenario and how manufacturers participating in sharing economy perform during COVID-19, no specific challenges and interventions that can assist manufacturers fight COVID-19 are presented. The final shortlist contained 56 relevant articles which were reviewed in full to be included in this paper. Some of the descriptive statistics are summarized and presented in Sect. 3.1.

## Findings

### Bibliometric insights

The review found that most studies report gross domestic product (GDP), trade volumes, and manufacturing outputs to encapsulate the tremendous external shock caused by the COVID-19 pandemic to the global economy. The GDP, worldwide, was on a collective decline, particularly in early 2020; the US (↓1.22%), Japan (↓0.85%), the UK (↓1.98%), Germany (↓2.22%), China (↓10.2%) and France (↓5.83%) were all demonstrating a downward trend (Cai & Luo, 2020; Gu et al., 2020; Luo et al., 2020). Several studies (Napoleone & Prataviera, 2020; Payne et al., 2021; Rapaccini et al., 2020) reveal that March 2020 was problematic for Italian manufacturing firms in comparison to other European countries. Italy’s industrial production decreased by 28.4%, and its stock exchange was on a high–low decline of 42%, adversely impacting Italian GDP (Rapaccini et al., 2020). Interestingly, some other figures for overall trade volumes are rather consistent, or even on a slight rise, for China and some parts of Northern Europe like Estonia; but foreign trade for Finland dropped by nearly 30% (Hilmola et al., 2020).

Around April 2020, the UK economy was preparing for recession, given its high reliance on manufacturing. Early figures suggest 70% of UK manufacturing firms reported loss of sales, only 11.7% firms operated at full capacity and 25% announced plans to make employees redundant, with the manufacturing output falling by 24.3%, recording the largest monthly decline since 1968 (Harris et al., 2020). Just a month later in May 2020, new figures show 41% of UK SMEs had to halt operations, out of which 35% feared permanent closure (Juergensen et al., 2020). Updated statistics are also available for the US and Taiwan; the second quarter of 2020 reports a sharp fall in US manufacturing production by 20.2%, followed by a GDP decline of 31.4% (Moutray, 2020), and Taiwan’s manufacturing report an 11% decrease in production value with an estimated 5.05% annual drop (Teng et al., 2021).

We also found research (Juergensen et al., 2020; Kim & Lee, 2021; Payne et al., 2021; Sahoo & Ashwani, 2020; Surianarayanan & Menkhoff, 2020) focused on COVID-19 complexities in the manufacturing SME context. About 70% SMEs in Italy and Europe, and 50% SMEs in Germany, report revenue losses due to COVID-19 (Juergensen et al., 2020). The figures captured in this section are mostly for the first and second quarters of 2020, and this is because most of the studies reviewed here were published in 2020. Out of the 56 publications included in this study, only 13 were published in the year 2021, with the remaining 43 published in 2020. Table 1 shows peer-reviewed journals and conferences with at least two publications of relevance to this review. Articles were also included from several other highly reputed journals (one article each), such as the *Harvard Business Review, Technological Forecasting and Social Change, International Journal of Operations & Production Management, Journal of Business Research, R&D Management*, and many more.

**Table 1:** Publications by journals and conferences

Journals/Conferences	Publications	Number of articles
Smart and Sustainable Manufacturing Systems	Diaz-Elsayed et al. (2020), Huang (2020), Kamarthi and Li (2020), Li (2020), Monostori & Váncza (2020), Terry et al. (2020), Wuest et al. (2020)	7
Sustainability	Hallstedt et al. (2020), Hilmola et al. (2020), Hoosain et al. (2020), Liu et al. (2021a, 2021b), Luo et al. (2020), Teng et al. (2021)	6
International Journal of Environmental Research and Public Health	Bragazzi (2020), Kim & Lee (2021), Manero et al. (2020), Ren et al. (2020)	4
Journal of Manufacturing Systems	Patel & Gohil (2020), Rajesh (2021), Tareq et al. (2021)	3
IEEE International Conference on Industrial Engineering and Engineering Management	Mantravadi et al. (2020), Okorie et al. (2020), Paucar et al. (2020)	3
Annals of Operations Research	Ivanov (2021a), Queiroz et al. (2020)	2
Technovation	Obradović et al. (2021), Tian et al. (2021)	2
Industrial Marketing Management	Elsahn & Siedlok (2021), Rapaccini et al. (2020)	2

Next, a detailed examination of the contents of all the shortlisted articles was undertaken. The insights from these studies were categorized based on frequently occurring keywords (such as, *digital*) including synonyms and phrases (such as, *Internet of Things*), and the research aim (such as, *impact of digital technologies on manufacturing*) to reflect on their relative importance. This allowed us to categorise articles by topic (Table 2), and this revealed that most studies focus on the role of digital technologies, including a focus on additive manufacturing and the issue of supply chain resilience.

**Table 2:** Publications by research topic

Research topic	Publications
Role of digitalization and IoT	Advincula et al. (2020), Belhadi et al. (2021), Bond III et al. (2020), Bragazzi (2020), Hallstedt et al. (2020), Harris et al. (2020), Hoosain et al. (2020), Javaid et al. (2020a), Kim and Lee (2021), Li (2020), Mantravadi et al. (2020), Mohammed & Isa (2021), Monostori and Váncza (2020), Payne et al. (2021), Tian et al. (2021), Yaqiong et al. (2020**)**
Supply chain management and resilience	Belhadi et al. (2021), Cai & Luo (2020), Handfield et al. (2020), Ivanov (2020, 2021b), Kamarthi & Li (2020), Li (2020), Linton and Vakil (2020), Paul & Chowdhury (2020a), Pinna & Lodi (2021),Queiroz et al. (2020), Rajesh (2021), Sharma et al. (2020), Terry et al. (2020)
Additive manufacturing	Advincula et al. (2020), Gu et al. (2020), Manero et al. (2020), Patel & Gohil, 2020), Tareq et al. (2021)
Aggregated impact on manufacturing	Harris et al. (2020), Hilmola et al. (2020), Luo et al. (2020), Moutray (2020), Obradović et al. (2021), Okorie et al. (2020), Rapaccini et al. (2020)
Impact on SMEs and MSMEs	Juergensen et al. (2020), Lu et al. (2021), Priyono et al. (2020), Sahoo & Ashwani, 2020), Surianarayanan and Menkhoff (2020)
Repurposing and reconfiguring	Elsahn & Siedlok, 2021), Liu et al., (2021a, 2021b), Napoleone & Prataviera (2020), Paul & Chowdhury (2020b
Digital servitization and transformation	Bond III et al. (2020), Liu et al. (2021a, 2021b), Payne et al. (2021), Tian et al. (2021)
Sustainable manufacturing	Huang (2020), Monostori & Váncza (2020), Teng et al. (2021)
Impact on environment	Diaz-Elsayed et al. (2020), Fankhauser et al. (2020)
Lean manufacturing	Ali (2021), Khoo && Hock (2020), Paucar et al. (2020)
Impact on workforce	Ren et al. (2020), Wuest et al. (2020)

Next, we focused on theories used by the shortlisted studies to explore the impact of COVID-19 in manufacturing (see Table 3). Most of these studies focus on investigating different aspects of supply chains. For instance, the *social capital theory* is used to study the relationship between digitalization, social capital, and supply chain performance (Kim & Lee, 2021). Additionally, with the pandemic presenting challenges around timely deployment of resilient assets, studies like Belhadi et al. (2021) and Ivanov (2021a, 2021b) employ the *supply chain resilience theory* to evaluate supply chain strategies. There is also evidence of the *theory of constructal law* being used to understand the makeup of supply chains and management-level decisions in the context of pandemic-related events (Handfield et al., 2020).

**Table 3:** Publications by theories

Academic theory	Publications
Social capital theory	Kim & Lee (2021)
Free cash flow theory	Teng et al. (2021)
Resourcing theory	Elsahn & Siedlok (2021)
Supply chain resilience theory	Belhadi et al. (2021), Ivanov (2021a, 2021b)
Resource-based view	Obradovi et al. (2021)
Location theory	Luo et al. (2020)
Leagile theory	Khoo & Hock (2020)
Constructal theory	Handfield et al. (2020)
Job demands-resources theory	Ren et al. (2020)
S-T theory	Tortorella et al. (2020)
Complexity theory	Rajesh (2021)

Another theory used to explain manufacturing firms’ decisions to adopt sustainable and resilient strategies in challenging contexts is the *resource-based view* (Obradovi et al.,2021). *Resourcing theory*, on the other hand, is used to explain the relationship between policymakers and collaborative innovation (Elsahn & Siedlok, 2021). We also noticed studies adopting a range of theories to study financial aspects; for instance, *free cash flow theory* is used to demonstrate the impact of financial flexibility on firm performance (Teng et al., 2021). There was also evidence of *location theory* being used to explain the importance of optimal firm location in terms of lowering costs of raw materials, labour, and freight (Luo et al., 2020). Another theory, which focuses on aspects of low cost and high quality, especially in volatile markets such as those induced by the pandemic, is the *leagile (lean and agile) theory* (Khoo & Hock, 2020).

There are further calls for research (surveys and case studies) to develop theories focused on strategies for supply chains and firm recovery in dire times, such as the COVID-19 pandemic (Paul & Chowdhury, 2020a). In their road to recovery from the pandemic, many firms are exploring the inclusion of digital technologies as a part of their strategy. There is a need for further theory development aimed at theorizing the interplay between organizational strategies and digital technologies (Priyono et al., 2020).

### Challenges in manufacturing emerging from COVID-19 complexities

#### Pre-existing challenges in manufacturing: better or worse since the pandemic?

Here, we discuss some of the challenges that manufacturers have struggled with since before the pandemic. Interestingly, while most of these challenges have intensified with the pandemic, one has been rectified to a certain extent. Firstly, there is the persistent problem of the skills gap and difficulties in recruiting a new generation of workers in manufacturing; most firms have an ageing manufacturing workforce (Harris et al., 2020; Moutray, 2020; Wuest et al., 2020). Although on the one hand, the pandemic has strained the labour market, making it difficult for manufacturers to find and retain a quality workforce; on the other, there are also new opportunities arising to train/upskill unemployed workers from the hard-hit service industries to make up for the current workforce shortage (Moutray, 2020).

Secondly, there is the challenge of finances required for incorporating digital technologies (Cai & Luo, 2020) that are now deemed critical for maintaining productivity and other downstream activities, such as marketing and sales (Juergensen et al., 2020). With COVID-19, investments in disruptive technologies have augmented; more manufacturers are incorporating digitized operations, but there are persistent worries around the return on investments (Surianarayanan & Menkhoff, 2020). Some studies report that digital struggles are much severe in an SME context, as preparing SMEs for I4.0 is extremely challenging when most of them (in the UK, at least) are still using I2.0 (Harris et al., 2020). The challenge of financial constraints is a longstanding concern, particularly for standalone manufacturing SMEs (Juergensen et al., 2020); these have only worsened with the pandemic, as SME budgets have become tighter and external financial support is not readily available.

Thirdly, financial burdens go beyond investments in digital technologies, because now there is an immediate need for a digitally-skilled workforce; this means manufacturers have to invest more in training (Huang, 2020; Kamarthi & Li, 2020). The industry has already recorded investment figures of around $26.2 billion in 2019 on training employees in new technologies (Moutray, 2020), and these are now expected to rise further. In addition to training, firms also have to devise incentives to keep the workforce engaged and interested in acquiring the necessary digital skills (Moutray, 2020).

Lastly, there are challenges created by the US–China trade war. The trade war in 2019 resulted in soaring global trade tensions, which have only multiplied with the pandemic (Cai & Luo, 2020; Okorie et al., 2020). Many manufacturers pulled out of China before the pandemic, but continued to rely on it for some intermediate goods (Handfield et al., 2020), and now, with the pandemic, trust between China and western nations is on a path of swift decline (Cai & Luo, 2020). The subsequent logistical issues concerning the import of manufacturing goods, combined with the challenges of reshoring, protectionism, and financial constraints in developing nations have only worsened (Cai & Luo, 2020).

#### Challenges related to lockdowns

The recent rise in fragmented economic activity, combined with the decline of vertically integrated firms have led to the rise of global supply/value chains (Juergensen et al., 2020). Most original equipment manufacturers and their tier 1 suppliers rely on international production bases and global supply chains (Handfield et al., 2020). Global markets have long remained the key to success for many of these manufacturers (Moutray, 2020); but national lockdowns cause logistical disruptions across borders and interrupt production networks and work, both upstream and downstream of the industrial chains (Cai & Luo, 2020; Tareq et al., 2021). The production nodes are underperforming and the logistic links are broken, making it clear that manufacturing is now fully exposed to supply shocks (Gu et al., 2020; Monostori & Váncza, 2020). Intensifying the complications is the unknown recovery timeline of these supply chains from COVID-19 shocks, which is, in turn, threatening the viability of international economic activities and interconnections (Cai & Luo, 2020; Kim & Lee, 2021; Pinna & Lodi, 2021; Terry et al., 2020) that are crucial for a stable global economy.

The industry is facing disruptions from the internalities and externalities of market turbulence, and the logistical uncertainty of product movements by land, air and sea (Khoo & Hock, 2020) is resulting in reduced capacity utilization (Juergensen et al., 2020). This is stunting key activities, such as the procurement of raw materials, and the import/export of key components. The restricted movement of goods, services and the workforce has, in instances, halted production altogether (Kamarthi & Li, 2020; Lu et al., 2021). Adding to these supply issues are problems such as flight cancellations, also resulting in rising costs for airfreight and haulage, and longer waiting times due to restricted road transport and increased commodity checking (Cai & Luo, 2020).

The most affected manufacturers are those that are heavily reliant on global supply chains, international labour and export-intensive operations (Harris et al., 2020). Matters are worse for manufacturers that have not diversified their suppliers (Hilmola et al., 2020; Linton & Vakil, 2020) and for those that rely on foreign low-cost suppliers to avoid expensive regional ones (Handfield et al., 2020). While relying on single and/or foreign suppliers may keep costs and other targets in check, the strategy backfires in crises such as the COVID-19 pandemic (Juergensen et al., 2020). Also impacted are those manufacturers that only account for their direct supplier, without monitoring the status of their lower-tier suppliers (Linton & Vakil, 2020). Supply-side shocks, thus, become inevitable, resulting in unsolicited economic commotion in the demand-side, also reflected in reduced disposable income and savings (Sahoo & Ashwani, 2020).

Another unique challenge of shutdowns/lockdowns is the fate of finished products, including food items with limited shelf lives, which are unable to reach the markets due to logistical disruptions (Diaz-Elsayed et al., 2020); these are generating piles of waste with nowhere to go, and the absence of recycling programmes for unused products is not helping the situation. Some studies including Harris et al. (2020) and Juergensen et al. (2020) find that lockdowns have created many challenges for UK manufacturers and European SMEs, with the supply side suffering from logistical disruptions and labour shortages, and the demand side hit by declining demands owing to the lack of customer confidence in the sustainability and performance of numerous global supply chains. Many manufacturing companies are buried under financial pressures, such as those in China; worse perhaps, many firms are suffering product delivery and supply chain pressures (Lu et al., 2021). Overall, raw material shortages and supply chain fractures during lockdowns have intensified the challenges of low rework rates, high operating costs and constricted cash flow, which are accumulatively obstructing full-scale production (Lu et al., 2021).

#### Challenges related to essential products and medical equipment shortages

The manufacturing industry was under tremendous pressure to meet the sudden increase in demand for critical medical equipment and associated paraphernalia, such as ventilators, surgical masks, gloves, testing swabs, face shields, sanitizers, respirators, oxygen valves, and other personal protective equipment (PPE) (Advincula et al., 2020; Bragazzi, 2020; Diaz-Elsayed et al., 2020; Hoosain et al., 2020; Liu et al., 2021a, 2021b; Napoleone & Prataviera, 2020; Patel & Gohil, 2020; Queiroz et al., 2020; Tareq et al., 2021; Wuest et al., 2020). The World Health Organization (WHO) stated that PPE production had to go up by 40% to meet the shortage in 2020 (Diaz-Elsayed et al., 2020). In addition to the medical supplies, manufacturers of specific non-substitutable essential items, such as hand sanitizers and toilet paper, also faced a massive surge in demand (Wuest et al., 2020).

It becomes clear from our review that the challenges that evolved thereafter are the first of their kind to hit the global manufacturing industry right at the onset of the pandemic in March 2020. The supply chain failure meant limited raw material availability, which coupled with reduced production capacity prevented manufacturers from fulfilling any of the above-mentioned demands (Paul & Chowdhury, 2020a, 2020b). As the situation evolved, the bullwhip effect enveloped the manufacturing industry, causing disruptions and putting integrated supply chain operations in jeopardy, and the challenges of immediate product shortages, logistical bottlenecks, and even overproduction became prominent (Cai & Luo, 2020; Handfield et al., 2020). Visibility across supply chains is generally limited, which when combined with such a bullwhip effect, leads to panic and, in the case of COVID-19, has caused further problems of inventory excess and stockpiling (Diaz-Elsayed et al., 2020).

Furthermore, as the pandemic peaked and passed through different waves, governments publicly appealed to non-medical businesses and organisations to share their supply chain capabilities and manufacturing capacities to meet the critical life-saving equipment shortages (Advincula et al., 2020; Elsahn & Siedlok, 2021). Those that are able to repurpose, producing such equipment are challenged with aspects of adhering to—medical safety and sterility protocols, design qualifications and other testing procedures (Advincula et al., 2020). Additionally, those that have not been able to repurpose identify the challenges of gaining the skills required for repurposing, the high costs involved, and other time constraints (Okorie et al., 2020).

#### Challenges related to safety protocols

Unlike other industries, in response to governments’ stay at home orders during the pandemic, manufacturing companies have not been able to move operations fully online and engage in remote working. Not much can be done where access to the production line, machinery, or laboratories is a must, or where specialist equipment cannot be used in a work-from-home setting (Juergensen et al., 2020). As a result, many manufacturers have had to either operate at limited capacity or terminate operations (Cai & Luo, 2020; Hilmola et al., 2020).

In addition to the challenges of remote working, safety guidelines from governments’ warranting social distancing in physical facilities are adding to manufacturers’ problems. Despite being a hands-on, physical industry, the need to social distance now requires manufacturers to reengineer production processes, identify and enable remotely operated procedures where possible, reconfigure workplace models, and restructure workforce management. While these align with safety measures, there are outstanding risks of poor responsiveness and reduced outputs (Moutray, 2020). Moreover, adhering to government restrictions and implementing physical changes to make manufacturing plants safety compliant involves substantial financial investments, increasing monetary burdens for manufacturers (Juergensen et al., 2020). All these issues are snowballing into prominent challenges of workforce shortages and poor production rates that are negatively impacting economic stability, and complicating supply chain management in manufacturing (Ivanov, 2021b; Kim & Lee, 2021; Lu et al., 2021).

#### Challenges related to workforce shortage and occupational dissatisfaction

Manufacturing firms have had to do much more than readjust their physical spaces to make work COVID-compliant. In particular, to enable remote working, as discussed above, manufacturers are having to employ a variety of digital technologies to support operations. A direct implication is related to workforce perception, where, specifically, low skilled or less educated workers begin to fear the possibility of being replaced by digital technologies. This perception may evolve into constant stress in the workforce and dissatisfaction in manufacturing as a career choice (Ren et al., 2020). Workforce shortages have been a prevalent issue in manufacturing, but are now becoming even more prominent, as there are shortages of people with traditional foundational skills combined with timely digital skills (Wuest et al., 2020).

In the race to employ people skilled in digital technologies, manufacturers cannot afford to lose those proficient in manufacturing skills. In a parallel vein, one study (Sahoo & Ashwani, 2020) explains the issue of the reverse migration of skilled manufacturing workers, which is having a negative impact on such a labour-intensive industry. Their study highlights how the manufacturing industry in India relies on migrant workers who acquire specific skills over the years. However, reduced operations due to COVID-19 has led to many workers moving back home to rural parts. Estimations from the International Labour Organization suggest disruptions in manufacturing have put almost half of the workforce worldwide at the risk of losing livelihoods (Koch et al., 2021). Concerns over losing their jobs (even as a possibility) is motivating them to look for other work. The current lack of stability in manufacturing is, therefore, creating dissatisfaction in workers, who are, then, less likely to return to work after lockdowns or temporary layoffs, causing skill shortages and further falls in industrial productivity (Sahoo & Ashwani, 2020). Moreover, the younger generation of men and women are finding it difficult to see manufacturing as a lucrative career opportunity. This further increases the risk of reduced numbers of skilled workers going forward. Some researchers (Harris et al., 2020) are of the view that the lack of substantial training programmes, internships, attractive salaries and other incentive-based frameworks is the reason for the loss and shortage of skilled labour.

### Management interventions for tackling COVID-19 challenges in manufacturing

#### Localizing and regionalizing production, value networks and supply chains

The localization and regionalization of supply chains are expected to be the new norm for manufacturing in the post-COVID period (Cai & Luo, 2020; Handfield et al., 2020). Some manufacturers are also exploring the possibility of adding new suppliers in multiple locations (duplication) to better manage supply chains (Moutray, 2020). Many studies are unanimously of the opinion that COVID-19 has both demonstrated and validated the importance and impact of localized manufacturing (Advincula et al., 2020; Tareq et al., 2021). For instance, its positive impact becomes evident in how countries managed medical equipment shortages by relying on domestic manufacturers to lead the production of supplies (Elsahn & Siedlok, 2021). More manufacturers are now seeking new suppliers and buyers within their home country (Lu et al., 2021) and are exploring the opportunities of localized production, i.e., reshoring (Harris et al., 2020; Moutray, 2020). By introducing changes with reshoring and similar initiatives in global supply chains, manufacturers are aiming to diversify their supply chains and manage their stocks better by assuming the benefits of proximity (Juergensen et al., 2020). Altogether, localized supply sources and regional value networks can be very useful for diversifying, mitigating risks to business continuity, controlling transaction costs, improving economies of scale, and increasing supply chain resilience (Belhadi et al., 2021; Kim & Lee, 2021).

#### Reconfigurability and repurposing

Most studies reviewed here (Cai & Luo, 2020; Elsahn & Siedlok, 2021; Ivanov, 2021b; Javaid et al., 2020b) discuss the rise of repurposing. Firms applied scalability and convertibility principles (Napoleone & Prataviera, 2020) to their smart manufacturing systems and logistical capacities to support the healthcare supply chain. Reconfiguring their industrial systems and machine tools enabled manufacturers to adapt their production lines and capacities to new demands, enabling repurposing (Monostori & Váncza, 2020; Napoleone & Prataviera, 2020). For instance, car manufacturers repurposed their lines to produce respirators (Advincula et al., 2020; Monostori & Váncza, 2020), and appliance manufacturers repurposed production lines of hairdryers and vacuum cleaners to produce ventilators (W. Liu et al., 2021a, 2021b). Following governments’ appeals to share supply chain capabilities and manufacturing capacities to meet critical life-saving equipment shortages (Advincula et al., 2020; Elsahn & Siedlok, 2021), the public and societies worldwide also stepped up as repurposed manufacturers. Individuals have used garage spaces and universities their resources and skilled staff/students (engineering teams) to repurpose (Advincula et al., 2020; Hoosain et al., 2020; Okorie et al., 2020; Tareq et al., 2021; Tian et al., 2021) to meet shortfalls in supply.

Interventions like these introduce manufacturing flexibility that enables demand and supply reallocation to manage changes smoothly in the production systems (Ivanov & Dolgui, 2020). Such temporary repurposing not only fulfilled the surging demand for medical supplies, but also, to some extent, made up for manufacturers’ lost demand of their normal production line (Juergensen et al., 2020). Moreover, in tackling the bullwhip effect, manufacturers have chosen to rearrange their capacities and targets; evidence suggests that many manufacturers used moderate operational optimization strategies, whereby they adjusted their targets to cope with the pandemic (Lu et al., 2021).

#### Coopetition and collaborative manufacturing

With COVID-19, coopetition – collaboration between competing businesses – is emerging as an effective strategy for supporting resourcing strategies (Elsahn & Siedlok, 2021) and preventing stock-outs (Hilmola et al., 2020). Many studies advocate coopetition for increasing supply chain resiliency, improving manufacturers’ service levels and safeguarding their reputations (Paul & Chowdhury, 2020b). Another output of coopetition is exaptation, whereby ecosystems evolve for the collective good in order to explore the possibility of pivoting a new function from an already existing one without additional developmental costs (W. Liu et al., 2021a, 2021b). It is also expected that coopetition may enable emergency sourcing of raw materials to allow production of some items in larger quantities, despite many manufacturers still operating at reduced capacity (Paul & Chowdhury, 2020b). Such competitor collaboration is aimed at bringing value for all stakeholders whilst fulfilling the critical demands of the hour (Rapaccini et al., 2020; Sharma et al., 2020). In addition, collaboration with trade unions/associations and other value chain stakeholders is also recommended by some studies (Napoleone & Prataviera, 2020; Rapaccini et al., 2020).

#### Lean and agile manufacturing techniques

Flexible strategies supporting the supply, demand and process functions can independently increase the flexibility and resilience of manufacturing supply chains (Rajesh, 2021). With operations centred on standardized processes despite limited resources, lean manufacturing is re-emerging as a flexible technique for improving the pandemic readiness of manufacturers (Abdallah Ali, 2021; Handfield et al., 2020; Paucar et al., 2020). Studies propose a combination of lean and agile systems—eagile manufacturing—that can help manufacturers stay ahead of their competitors even in such disruptive times (Khoo & Hock, 2020). This technique can help tackle COVID-induced volatility in markets and consumer needs by responding to unstable demands downstream, and offering level scheduling upstream (Khoo & Hock, 2020). Many studies recommend agile smart supply chain planning, potentially to supress supply and demand volatility whilst recalibrating and optimizing supply chain operations (Cai & Luo, 2020). In addition, lean approaches can achieve value at the low cost while maintaining stable demands, including an agile paradigm for aptly responding to demand fluctuations in product lines (Okorie et al., 2020). For instance, a study (Abdallah Ali, 2021) employed process optimization embedded in lean manufacturing across an aluminium factory and achieved significant reduction in the need for labour (down by 50%), including noteworthy cost savings, rendering the factory pandemic-ready.

#### Digital technologies

Industry 4.0 (I4.0) and digital technologies in manufacturing were already on the rise (Juergensen et al., 2020) prior to COVID-19. Now digital technologies are considered key for long-term resilient manufacturing (Kamarthi & Li, 2020; Queiroz et al., 2020; Sharma et al., 2020). Digitalized supply chains and networks reduce design complexities and improve connectivity and resource flow, which help in managing existing strategic relationships whilst identifying new possible relationships (Kim & Lee, 2021). I4.0 manufacturing execution systems can expedite manufacturers’ response to severe market disruptions, such as the one caused by COVID-19, including adverse changes in market demand, material flows, replenishment and composition to enable manufacturing flexibility (Mantravadi et al., 2020). Our review suggests, COVID-19 has fast-tracked the ongoing trend of digitalization, with more manufacturers now realizing the urgency of investing in new technologies (Belhadi et al., 2021; Mantravadi et al., 2020; Moutray, 2020; Rapaccini et al., 2020). Most firms are focusing on IoT-based systems touted for their potential to maintain agility and visibility across networks (Kim & Lee, 2021; Sharma et al., 2020; Wuest et al., 2020). Industrial IoT (IIoT) can connect factories to minimize production risk from plant closures (Li et al., 2020).

Manufacturing will soon be characterized by automated processes, advanced manufacturing, and digital customer interactions (Javaid et al., 2020b; Juergensen et al., 2020; Terry et al., 2020). Many studies have vouched for Artificial Intelligence (AI) and other digital technologies to improve social capital, and increase supply chain productivity (Kim & Lee, 2021). Digital technologies also help align human capital and environmental resources to improve product lifecycle predictions (Diaz-Elsayed et al., 2020). In extending the discussion on AI, some studies also investigate the potential of robotics. The increased human–machine interaction supports social distancing and expedites manufacturing (Javaid et al., 2020b). This allows industries and individuals to carry out both commercial and non-commercial operations, sometimes with increased precision; for instance, the use of AI and robotics in healthcare (Cai & Luo, 2020), (Monostori & Váncza, 2020). Another advantage of AI is that it enables continuous monitoring of global suppliers (Linton & Vakil, 2020). Investing in supplier monitoring can help control inventory, monitor deviations, forecast changes, track and trace raw materials, and fast-track responses (Linton & Vakil, 2020). Other technologies like Big Data Analytics (BDA) and digital twins coupled with predictive engineering are also very effective in transmitting real-time information to stay up to date on supply chain activities in uncertain situations (Belhadi et al., 2021; Mantravadi et al., 2020; Okorie et al., 2020). Such options can minimize capacity losses, and reduce societal health risks (by overcoming complexities of travel restrictions and social distancing) (Li et al., 2020) to mitigate production-related COVID-19 disruptions.

Additive manufacturing, such as 3D printing, has also received tremendous interest from researchers (Bragazzi, 2020; Monostori & Váncza, 2020; Napoleone & Prataviera, 2020), as it enables real-time short-run production aimed at quickly fulfilling critical demands, while keeping waste to a minimum (Manero et al., 2020; Patel & Gohil, 2020). Unpredictable demands in turbulent times are best tackled by employing technologies such as 3D printing as they enable flexibility (Liu et al., 2021a, 2021b); for instance, 3D printing is reducing dependence on normal supply chains to empower localized production of critical medical equipment (Advincula et al., 2020). Use of 3D technologies (in addition to IoT, AI, machine learning, robotics, Big Data) aligns with the United Nations sustainable development goals in potentially offering solutions to most societal problems, including COVID-19 (Hoosain et al., 2020). Many 3D printing companies are publicly sharing their manufacturing technology, so corporations and individuals with enough resources can use it to produce necessary parts to be used in treating COVID-19 patients (Tareq et al., 2021). Global open-source designs are also available and these enable the use of 3D printing for designing and manufacturing PPE and medical equipment at a large scale (Hoosain et al., 2020; Javaid et al., 2020b; Liu et al., 2021a, 2021b). Supporting equipment, such as drones (for surveillance, lockdown enforcement, and even spraying disinfectants to aid sanitization) and hands-free door openers are being produced using this technology (Patel & Gohil, 2020).

#### Servitization and service provision

Many manufacturers have been rapidly turning to servitization—a transformation process, whereby companies shift focus from ‘creating value by producing/selling a product’ to ‘creating value by delivering a service enabled by that product’ (Kapoor et al., 2021a, 2021b). Having the reputation for stabilizing business operations across manufacturing firms during turbulent times, some studies propose servitization as a tactic to help manufacturers redesign their offerings, so they can find alternative ways to recover from disruptive events (Okorie et al., 2020; Rapaccini et al., 2020; Tian et al., 2021). Studies (Rapaccini et al., 2020; Tian et al., 2021) suggest a combination of servitization and digitalization, i.e., digital servitization that employs IIoT (also referred to as *smart servitization*) and other smart connected products to deliver advanced services to customers during COVID times. Manufacturers offering advanced services via platform-based servitization models present a higher likelihood of performing better than those limited to basic services in disruptive situations like the pandemic (Tian et al., 2021).

#### Government policies

Government policies are expected to play a significant role, with the supply, demand and environment policy types now enforced to tackle COVID-19 challenges (Cai & Luo, 2020). Policymakers are being expected to play a role in supporting resourcing strategies, mostly by acting as communicators and negotiators to enable coordination, cooperation, and knowledge sharing between competing manufacturers (Elsahn & Siedlok, 2021). In fact, given the more cooperative nature of current manufacturing, some studies have contemplated the need for policies in a post-COVID era that can support cooperative (coopetition) manufacturing (Monostori & Váncza, 2020). Furthermore, policies are needed for increasing manufacturing competitiveness, specifically for those businesses that are restructuring supply chains and considering on-shoring (Moutray, 2020). Support policies for restoring production activities and manufacturing supply chains are also on the horizon (Lu et al., 2021).

In addition, some studies (Lu et al., 2021) find that manufacturing industries are more inclined towards tax preferences and employment subsidies. Policies should thus focus on long-term subsidized digitalization-specific loans, funding for training and apprenticeships, financial aid to support company growth, and digital advisory support available for supply chains (Harris et al., 2020). In addition to the advisory, specific policies can be aimed at capacitating academic institutions with training programmes and certifications, to upskill the workforce in rendering them digitally fit (Diaz-Elsayed et al., 2020; Kamarthi & Li, 2020). In further empowering the workforce, propositions have been made to introduce interest-free capital from governments to account for fixed costs in the industry, including wages (Sahoo & Ashwani, 2020). There is some evidence to suggest stimulus measures and policies focused on labour can effectively protect employment and lower the liquidity crunches for boosting the economy (Juergensen et al., 2020; Sahoo & Ashwani, 2020).

However, one size does not fit all; SMEs are most vulnerable and policies on internationalized networking and demand-oriented product and marketing innovations will be most effective for them (Juergensen et al., 2020). Targeted policies supporting entry into international markets would be most useful for standalone SMEs, and specialized SMEs would benefit from stronger local networks promoting the inclusion of their customers (multinational businesses) in the territory (Juergensen et al., 2020). Additionally, some recommendations demand policymakers’ attention to specifically tackle issues of connectivity across borders and rising transportation costs (Hilmola et al., 2020). A more generic recommendation is for systematic macro-guidance from governments to oversee the execution of targeted policies (Luo et al., 2020).

## Discussion

### Summarizing the findings

This review identifies several challenges under the five overarching categories—pre-existing challenges in manufacturing that are now worse with the pandemic, those related to lockdowns, safety protocols, shortages of essential products, and a shortfall in the skilled workforce. These challenges directly address RQ1. In addition, in response to RQ2, seven different categories of management interventions have been extracted—localizing and regionalizing production, value networks and supply chains, reconfigurability and repurposing, coopetition and collaborative manufacturing, lean and agile manufacturing techniques, digital technologies, servitization and service provision, and government policies. These interventions have now been mapped against the specific challenges in Table 4.

**Table 4:** Mapping challenges with management interventions

COVID-19 Challenges in manufacturing	Management interventions	Problems with management interventions
*Pre-existing challenges in manufacturing*		
Ageing workforce & workforce shortage Lack of incentives to motivate workforce High costs of digital technologies Financial challenges for SMEs Trade war complexities	Digital technologies/IoT	High training costs of digital upskilling High costs of adopting digital technologies
	Government policies	Lack of procedures for organizing orders & contracts to keep the industry afloat Problems with global supply chain coherence & coordination Lack of SME-specific support policies for international networking & demand-oriented product & marketing innovations
*Challenges related to lockdowns*		
Underperforming production nodes Reduced revenues Closed production facilities Fractured supply chains Reduced capacity & production High operating airfreight & haulage costs Delayed & restricted freight transport Increased commodity checking at borders Overreliance on single & foreign suppliers Low rework rates	Localizing/regionalizing production, value networks & supply chains	Time consuming to set up new networks High costs of making new suppliers compliant & proficient in necessary skills, qualifications & certifications Developing countries at risk of export restrictions, higher tariffs, countervailing duties & regulatory protectionism
	Government policies	No protection from policies for businesses relying on suppliers in foreign countries Tax cuts based on work resumption, no protection from lockdowns & restrictions Lack of protection from liquidity crunches
	Digital technologies/IoT	Digital divide & lack of technological readiness (missing infrastructure) SMEs still stuck at I2.0 Lack of internal expertise (supply chain managers, etc.) on digitalization Lack of coordination amongst supply chain stakeholders
	Servitization	Complications of readjusting KPIs quickly Penalties of missing service response time & uptimes, foundational to service provision Non flexible contractual agreements Outcome risk transferred fully onto providers & suppliers
	Lean & agile manufacturing	None recorded in research reviewed herein
*Challenges related to essential products & medical equipment shortages*		
Bullwhip effect Supply chain failure Raw material shortages Logistical bottlenecks Overproduction Inventory excess Stockpiling Limited supply chain visibility	Reconfigurability & repurposing	Lack of guidance on regulatory aspects, clinical safety, certification, standards and procedures for repurposing, using techniques like 3D printing High equipment costs – expensive strategy Time consuming Legalities & patent issues Insufficient human capital Increased plastic with overproduction of medical equipment causing environmental concerns
	Government policies	Most policies are a short term fix; lack of long term solutions
	Digital technologies/IoT	High costs of equipment, such as those required for 3D printing Adverse impact on environment—carbon footprint and excessive plastic
	Coopetition &collaborative manufacturing	None recorded in research reviewed herein
	Lean & agile manufacturing techniques	None recorded in research reviewed herein
*Challenges related to safety protocols*		
Inability to move all operations online Remote working limiting operations Social distancing warranting major reconfiguration of workplace models Major restructuring of workforce management Poor responsiveness & reduced outputs Complications in supply chain management	Digital technologies/IoT	Lack of digital expertise, limiting ability to upgrade as digital trends change Digital inequality between manufacturer and supply chain stakeholders hindering seamless operations Prone to cyber attacks; hacking, power outage and cyber warfare are active concerns Issues of enterprise data privacy
	Government policies	Lack of policies for managing cross border data Lack of subsidized digitalization specific loans
*Challenges related to workforce shortage & occupational dissatisfaction*		
Lack of work leading to reverse workforce migration Dissatisfaction in manufacturing career Lack of up to date skills Fall in industrial productivity	Digital technologies/IoT – Digitally train/upskill both current & unemployed workers from other hard hit industries	Workforce perceptions; fear of being replaced by digital technologies Lack of knowledge initiative to educate workforce that its intuitive problem solving skill on the shop floor is the key to successful digital operations Lack of training programmes, internships & attractive salaries to motivate & retain workforce
	Government policies	Absence of interest free capital from governments that can account for fixed costs in manufacturing, such as employee wages Lack of policies focused on employment protection

Most studies recommend the use of digital technologies as a management intervention for tackling COVID-19 complexities. While many broadly discuss these across the topics of IoT and IIoT or I4.0 (Belhadi et al., 2021; Mantravadi et al., 2020; Rapaccini et al., 2020), a significant number of other studies identify specific technologies such as AI, virtual reality, machine learning, robotics, blockchain, Big Data, cloud-based technologies, drones, 5G Radio Frequency Identification, and e-learning, as suitable platforms for supporting continuous production (Bragazzi, 2020; Cai & Luo, 2020; Hoosain et al., 2020; Kamarthi & Li, 2020; Mohammed & Isa, 2021; Monostori & Váncza, 2020; Okorie et al., 2020; Paul & Chowdhury, 2020a; Ren et al., 2020; Sharma et al., 2020; Yaqiong et al., 2020). Specific interest from a significant number of researchers also goes into exploring the possibilities of additive manufacturing, such as 3D printing, for mitigating the burdens of COVID-19 (Bragazzi, 2020; Monostori & Váncza, 2020; Napoleone & Prataviera, 2020). Using such technologies enables data sharing and scenario planning, and increases transparency; these together help manufacturers to anticipate/mitigate risks, intelligently forecast customer needs, and ascertain business continuity amidst a crisis (Cai & Luo, 2020). Overall, researchers believe the recent crisis will not only accelerate the adoption of digital technologies, but will also force the industry to experiment with more robust solutions, such as everything-as-a-service and servitization (Bond III et al., 2020; Moutray, 2020), capable of withstanding pandemic-level events.

What also becomes apparent from the review is the uncertainty around the longevity of manufacturers’ relationship with digital technologies—studies ponder about the use of such technologies merely being a short-term objective for manufacturers to stay operational in COVID-times (Kamarthi & Li, 2020; Monostori & Váncza, 2020; Terry et al., 2020; Wuest et al., 2020). Some researchers suggest that COVID-19 is forcing SMEs and other traditional manufacturers to recognize the benefits of digitalization, and that this behaviour indicates permanent intentions of digital transformation (Surianarayanan & Menkhoff, 2020). However, others believe that, although businesses see the short-term benefits, they remain suspicious of the usefulness of such technologies post-pandemic (Priyono et al., 2020). Then, there are also studies (Okorie et al., 2020) concluding that, for manufacturers to reap the full potential of I4.0 technologies, long-term digital commitments and investments are needed. Similar views exist around servitization (Rapaccini et al., 2020)—the pandemic is motivating manufacturers to alter business models so they can accommodate such technologies, but whether these shifts and transformations are here to last remains to be seen.

### Effectiveness of proposed management interventions

The findings demonstrate how manufacturers are intervening to address underlying weaknesses in manufacturing operations. We compare researcher views on such interventions across the 59 shortlisted studies to assess their effectiveness. As derived from this review, Table 4 lists the COVID-19 challenges in manufacturing, identifies the specific interventions recommended for managing these challenges, and pinpoints any problems associated with the execution of these interventions.

Out of the seven management interventions (Sect. 3.3) outlined in this review, researchers are in agreement and offer similar views for two of them: *coopetition and collaborative manufacturing*, and *lean and agile manufacturing techniques*. However, there are contradictory views for five of the interventions.

#### Contradicting views on localized/regionalized networks as a management intervention

Many researchers advocate the effectiveness of reshoring, localization and regionalization (Advincula et al., 2020; Kim & Lee, 2021; Lu et al., 2021; Tareq et al., 2021) to improve supply chain resiliency; but some (Handfield et al., 2020) highlight the associated difficulties of setting up new suppliers in local regions and alternative countries. It can be a long process (up to five years) for supply chains characterized by specialization and customization, as they will have to be re-evaluated against new criteria (Juergensen et al., 2020). Manufacturers would have to deal with the new challenges of reorganizing coordination between old and new players in the supply chain, ensuring adequate skills, finance specialisation and certifications were in place, and passing new tariff checks—all of which makes supplier replacement rather complex (Juergensen et al., 2020). In addition, production reshuffling and the restructuring of supply chains are also risks to global manufacturing distribution (Pinna & Lodi, 2021). It can put developing countries with insufficient capacity in a disadvantageous position, because preferential trade agreements will be readjusted, making them vulnerable to export restrictions, higher tariffs, countervailing duties, and regulatory protectionism (Pinna & Lodi, 2021).

#### Contradicting views on reconfiguring and repurposing as a management intervention

Globally stunted production and the rising demand for manufactured medical paraphernalia has resulted in additive manufacturing, such as 3D printing, filling the gaps between interrupted manufacturing cycles via repurposing (Manero et al., 2020). While research approves repurposing (Elsahn & Siedlok, 2021; Ivanov, 2021b) as an effective technique for tackling the extremities, it is not without flaws. As policies are being directed at increasing localized inventories of medical supplies (Handfield et al., 2020), many manufacturers have stepped up to repurpose, but they find it to be an extremely challenging task (Okorie et al., 2020). Manufacturers are not only having to fulfil the abnormally high demand for medical equipment and supplies in record time, but they also have to deliver these globally in some cases (Khoo & Hock, 2020). Problems of large variations in the quality of produced goods, regulatory aspects, inspections related to quality and clinical safety, certifications, lack of procedure and standards have been identified as outstanding issues associated with repurposed solutions employing 3D printing (Advincula et al., 2020; Bragazzi, 2020). Techniques like 3D printing can be linked to several other challenges of legalities and patent issues, ethical issues involving humans in testing medical products, and the durability of the products (structural issues) manufactured using them (Advincula et al., 2020). These techniques are expensive and time-consuming, and some scholars are of the opinion that they are temporary solutions (Advincula et al., 2020; Manero et al., 2020; Okorie et al., 2020).

Poor resource allocation is yet another barrier to effective repurposing (Okorie et al., 2020). While some studies (Manero et al., 2020; Surianarayanan & Menkhoff, 2020) suggest the use of pre-validated design files, updated guidance from regulatory institutions, sufficient human capital (local networks, community engagement, universities and council support for easy access to vetted equipment), others (Okorie et al., 2020) suggest employing benchmarking, so manufacturing can adapt best practices and process improvement strategies from other similar industrial sectors. Lastly, while repurposing has now enabled quick production of large amounts of medical supplies, massive amounts of plastic waste is being generated, given abundances of gloves, shields, robes and masks. Researchers are questioning the impact of such overproduction on the environment (Diaz-Elsayed et al., 2020).

#### Contradicting views on digital technologies as a management intervention

While many recommend digital technologies to increase manufacturers’ responsiveness to COVID-19 challenges, researchers are also cautious about employing such technologies for several reasons. Here, we segregate a comprehensive list of such reservations:

Firstly, security breaches are an active concern for manufacturers employing digital technologies (Sharma et al., 2020). Manufacturing recently recorded a sharp increase in cyber security incidents (Goettl, 2021). Many sites employ automation with the workforce situated offsite, but connected to shop floors via digital technologies; this is leaving manufacturing sites open to cyber-attacks (Diaz-Elsayed et al., 2020). Mostly, manufacturers do not have the required level of digital expertise, which makes them vulnerable to issues of hacking, power outage and cyber warfare (Rapaccini et al., 2020). Moreover, the use of I4.0 implies manufacturers possess large amounts of trade data and privacy information; this makes enterprise data privacy another challenge for most, as they have to manage national and cross-border data, whilst complying with national/international policy structures (Yaqiong et al., 2020).

Secondly, researchers acknowledge that digital technologies create new opportunities, but they also warn manufacturers about having to continuously innovate (Obradović et al., 2021; Wuest et al., 2020) and constantly upgrade technologies to stay up to date with trends, formulate strategic alternatives, and take risks in achieving competitive advantage (Kim & Lee, 2021). Just as important is the organizational acceptance of digitalization. Research is very clear about the advantages of technologies, such as AI and robotics (Holland et al., 2021; Mohammed & Isa, 2021), but their application and purposefulness can be jeopardized if supply chain managers do not adequately understand digitalization (Kim & Lee, 2021). In addition, all the supply chain stakeholders also have to be on board to collaborate and accelerate the seamless application of digital technologies across manufacturing operations (Belhadi et al., 2021).

Thirdly, there is the issue of digital divide, centred on the technological readiness of manufacturing organizations and their various stakeholders across the globe. While employing digital infrastructure is effective for improving supply chain visibility and tackling other adversities (Kim & Lee, 2021; Sharma et al., 2020), they are not entirely viable for, or accessible by, all parties. Not all firms have the same level of technological or digital readiness (Rapaccini et al., 2020; Sharma et al., 2020). This problem is prevalent across most SMEs, as the high costs associated with employing new technologies hinder the technological readiness of SMEs (Surianarayanan & Menkhoff, 2020). Also, digital inequality between developing and first world countries in accessing something as basic as the internet cannot be ignored either (Hoosain et al., 2020). For manufacturers with suppliers/stakeholders in developing countries with poor ICT infrastructures, including a lack of IT skills/competencies, realizing the full potential of these technologies can become difficult (Mohammed & Isa, 2021).

Fourthly, a direct consequence of increased digitalization with COVID-19 is that data centres are operating at full capacity. Evidence points at a critical environmental challenge because of the large amount of power (200 terawatt hours) consumed by these data centres resulting in carbon emissions. These levels are only expected to rise in the near future (20% of global carbon emission by 2030) (Hoosain et al., 2020). Thus, employing digital technologies, although a very promising intervention, is to a certain extent, having a negative impact on climate change.

Finally, researchers discuss the dangers of dated employee skills that are swiftly being replaced by automation and robotics (Harris et al., 2020; Kamarthi & Li, 2020). While such digital technologies increase the efficiency of business operations, some studies look at the issue from a different perspective: that training shop floor operators is not given due attention in the process of digital transformation. The argument is that the human workforce is the key to maintaining the smooth workflow and processes on shop floors (Wuest et al., 2020). The need for humans, given their intuitive problem-solving, is making a comeback, despite industry’s move towards heavily automated processes (Monostori & Váncza, 2020). A combination of a human workforce and digital technologies is essential for achieving operational efficiency in manufacturing operations. There is an immediate need for upskilling labour, so that workers are proficient in the use of digital technologies and also of cyber security, so manufacturers can explore the full potential of digital technologies.

#### Contradicting views on government policies as a management intervention

The COVID-19 situation has exposed the issue of missing governance structures (Bond III et al., 2020). Although the studies reviewed herein recognize the importance of policies, their poor execution is criticized by many. Most of the support policies which helped restore production activities and supply chains across the industry are a short-term fix, and manufacturers still need long-term solutions (Lu et al., 2021). Research also criticizes some policies such as tax cuts, which are primarily based on resuming work, followed by income generation. However, if restrictions in the pandemic prevent employees from returning to work, no orders are fulfilled and no revenue is generated; more simply, tax cuts did not suffice as a solution in the early COVID period (Lu et al., 2021). Studies have also criticized the UK and US governments’ minimal efforts in organizing their orders and contracts to keep the industry afloat (Elsahn & Siedlok, 2021).

In a different vein, an overreliance on foreign suppliers can make manufacturers hostage to the government policies of another country (Handfield et al., 2020). This is because variations in government policies across countries impact different aspects of coherence and coordination in manufacturing supply chains; researcher are debating the shift to local strategy (Harris et al., 2020).

#### Contradicting views on servitization as a management intervention

Moving onto servitization, most researchers are focused on exploring the benefits of growth and profitability that it brings for manufacturers (Okorie et al., 2020; Rapaccini et al., 2020; Tian et al., 2021). However, such outcome- and performance-based solutions can severely backfire (problems in meeting service response time and uptimes that such services are foundationally based on), as these (a) customized offerings can prevent customers from acquiring solutions from elsewhere when manufacturers struggle to redeploy resources and (b) contractual agreements lack the flexibility required in a crisis, making it complex to readjust the pre-agreed KPIs at a swift pace (Bond III et al., 2020). More interestingly, the review finds studies take starkly different perspectives on the nature of such service contracts. Some researchers show service contracts in a positive light to suggest managers can package them as fully risk-free, guaranteed-result contracts to cover fees and rents in the event of COVID disruptions (such as lockdowns and supply chain complexities) (Rapaccini et al., 2020). Other researchers look at them as adversities—uch contracts transfer most of the outcome risk onto the providers and suppliers, who are likely to be burdened with excessive costs and meagre revenues (Bond III et al., 2020).

### Research agenda

In order to support the manufacturing industry recover fully from the COVID-19 pandemic and be ready for future crises, academics should explore, investigate and scrutinize numerous topics. This review identifies several such avenues that need due attention from research communities.

#### Short- and long-term impacts of COVID-19 on manufacturing

Existing studies propose the need for further research on the short- and long-term impacts of the pandemic on major economies. Currently, a limited number of studies (Cai & Luo, 2020) explore the initial impact of the pandemic in manufacturing, and also study the aftershock. To understand more in this context, studies (Juergensen et al., 2020) recommend establishing a distinction between the immediate effects of COVID-19, specifically those related to the world going into a global lockdown during April–June 2020, and its long-term effects. Some researchers recommend a customer focus to learn how customers’ goals evolve during a crisis in the context of an immediate response versus the long-term change (Bond III et al., 2020), while others prefer a business focus, particularly in understanding how short-term digitally-enabled solutions can be aligned with long-term benefits for manufacturing organizations (Wuest et al., 2020). Evaluating such short- and long-terms impacts will shed light on the viability of manufacturing supply chains (Ivanov, 2020), and explain the revenue and value returns in the context of mitigating COVID-19 risks.

#### Policies focused on economic recovery for manufacturing, post-pandemic

As governments work on policies focused on the restoration of the economy, a broader line of research is the scrutiny of national and regional recovery roadmaps laid out specifically for manufacturing. Future research could investigate geographic nuances to establish how manufacturers could prepare to manage their operations across borders (Okorie et al., 2020). Moreover, this research would enhance our understanding of geographic variations on the impact of the COVID-19 recession on manufacturing and its subsectors (automobile, aviation, machinery and equipment, computers and more) (Harris et al., 2020). For instance, the uptake of advanced manufacturing could be higher in one region, or the technology readiness of another may be better – meaning their responses to the recession will vary. Overall, the bigger question is how can policymakers ensure that any future disruptive events will not create the same operational hurdles (Abdallah Ali, 2021) as those experienced with the COVID-19 pandemic

#### Sustainable reconfiguring and repurposing

Our review highlights the concept of reconfiguring and repurposing as a prominent intervention that directly helped address the issue of life-critical medical shortages. Although much is known of their benefits, there is not enough evidence to elaborate on the complexities of such reconfiguring and repurposing. How simple or complex is it for manufacturers to do so? While studies do offer insights on the difficulties associated with the use of some digital technologies, like 3D printing that have played a major part in repurposing, there is no dedicated research explaining the benefits and caveats (for manufacturers) of reconfiguring production lines over a period of time, answering questions such as: is this a sustainable option? How does it impact a manufacturing business?

There are dedicated calls for research to study how the reconfigurability theory and the dynamics capabilities theory are linked, to draw conclusions on reconfigurability across supply chains (Napoleone & Prataviera, 2020). More specifically, research should ask: what can be done to ensure the success of cross-sectoral supply chains in terms of complexity and speed of switching production lines for the sake of repurposing (Ivanov, 2020)? Such insights would clarify the link between the organizational capacity to change operations efficiently and develop new resources, and the actions required at different stages of manufacturing (Napoleone & Prataviera, 2020). Evidence suggests accelerated innovation is just as important as technologies for increasing manufacturers’ readiness to reconfigure and repurpose; however, this line of research is mostly available in the context of internal processes rather than external collaborations like coopetition (Liu et al., 2021a, 2021b). Future studies could explore the links between rapid responsiveness and accelerated innovation in exaptation to divulge insights on an ecosystem evolution that can potentially produce outcomes for the collective good to combat complexities triggered by disruptive events.

#### Network and supply chain recovery in manufacturing

Several studies (Juergensen et al., 2020; Linton & Vakil, 2020; Monostori & Váncza, 2020; Moutray, 2020; Paul & Chowdhury, 2020b; Sharma et al., 2020) suggest a common need for developing the knowhow to build resilient supply chains; but resilience is a rather complex notion to measure (Rajesh, 2021). Nevertheless, a solution might be to explore the relationship between supply chain resilience and factors such as efficiency, complexity, and the impact such resilience has on the environment. This information would allow researchers and practitioners to establish the trade-offs between resilience and these other factors (Monostori & Váncza, 2020; Rajesh, 2021). Future research should also focus on the difference between dynamic and evolutionary supply chain operations, and delve deeper into flexible supply chain contracts for reducing risks and expediting recovery to improve immunity to disruptive events in the future (Handfield et al., 2020; Linton & Vakil, 2020; Moutray, 2020; Rajesh, 2021). Another avenue for research is the development of contingency strategies to support manufacturers in risk recovery during disasters (Handfield et al., 2020). Additionally, very little is known about the production recovery models applicable for wide-scale disruptive events (Paul & Chowdhury, 2020a).

Some studies suggest the need for more stakeholder-inclusive research. For any network to function at maximum efficiency, it is essential that stakeholders are synchronized and there is uninterrupted information flow and visibility across the network (Monostori & Váncza, 2020). Insights from such research could also help in interpreting the various stakeholder-dependency relationships that come into effect as supply chains change in response to emergency situations like the pandemic (Hilmola et al., 2020). These dependency relationships are prominent in collaborative activities, so researchers should investigate how coordination between solution providers and customers is affected; i.e., what response protocols and governance mechanisms would be most effective during a pandemic (Bond III et al., 2020). Overall, more research on the topics of production recovery and supply chain recovery would add insights to help practitioners restructure supply chains, optimize operational strategies, and deliver greater value to everyone participating in those value chains.

#### Impact on the environment

Existing research is conflicted in discussing matters of manufacturers’ rapid response involving the inclusion of digital technologies and the impact this has on the environment (Diaz-Elsayed et al., 2020; Fankhauser et al., 2020; Hoosain et al., 2020; Monostori & Váncza, 2020; Patel & Gohil, 2020). While some (Harris et al., 2020; Monostori & Váncza, 2020) propose the adoption of digital practices and restructuring supply chains to account for transport-related carbon emissions, others (Fankhauser et al., 2020) find most emerging economies are less ready than leading countries (South Korea, Taiwan and China) for a zero-carbon recovery. More research is needed to understand the trade-off between digitalization and its carbon footprint. More specifically, given the many positives of digital manufacturing, research needs to investigate what can be done to keep environmental impacts of digitalization to a minimum. An additional line of research is the readiness of sectoral world economies in supporting a zero-carbon footprint. Manufacturing, globally, is one of biggest industries contributing to the green economy with growing demand for clean and green products, linked to national prosperity (Fankhauser et al., 2020). Thus, changes in the environment are highly likely to affect society and economies at large (Diaz-Elsayed et al., 2020); and so, future research should invest in exploring the accumulative impact of manufacturers’ COVID-19 response on the environment, embedded in the overarching aspects of society and national economies.

#### Data availability and utilization in a pandemic context

Manufacturers acknowledge that one of the outcomes of employing digital technologies is the large influx of data. However, how this data can be processed to be put to practical use still remains a challenging question (Moutray, 2020; Yaqiong et al., 2020). On the other hand, there is also the issue of a lack of data; for instance, in applying robotics to assist in urgent care, machine learning is key to program decision-making, sensing and adapting, but a lack of data on circumstances, such as emergencies during a pandemic that have not been observed and recorded previously, make this difficult (Holland et al., 2021). More research is needed to understand how such data and/or circumstances can be simulated to enable the application of automated technologies. Such insights will help us predict challenges, such as shortages, and assist in preplanning responses in order to avert crises (Queiroz et al., 2020).

#### Benefits of flexible manufacturing

In the interest of incorporating increased flexibility and responsiveness during a pandemic-level event, more needs to be known about the dos and don’ts of localization and regionalization. Upcoming research could explore the future of global sourcing to reveal what manufacturers can do to reduce their reliance on single/fewer suppliers (Handfield et al., 2020), with a focus on the need to diversify and limit the risks of monopsonistic situations (Juergensen et al., 2020). Studies, for instance, could look into clarifying how local and lean production systems could offer respite for struggling manufacturers (Handfield et al., 2020). Some researchers (Rajesh, 2021) advocate the suitability of flexible options, such as just-in-time (JIT) manufacturing, while others (Diaz-Elsayed et al., 2020) prefer sustainable production, as this has the potential to structure both material and data flows as part of a long-term framework, leading to higher supply chain resiliency. Future research could evaluate how sustainable production and JIT measured against COVID-19 complexities.

#### Managing demand fluctuations of essential items in a pandemic context

Research on managing the supply of high-demand, non-substitutable essential products (toilet paper, for example) and high-demand panic purchase items (for instance, foods with longer shelf lives, such as milk powder) during a crisis, is extremely scarce and only available in the post-disruption context (Cai & Luo, 2020; Paul & Chowdhury, 2020b). Further insights on the topic are expected to help manufacturers tackle the bullwhip effect (Handfield et al., 2020) and manage demand surges of such commercial products during unprecedented times. More inclusive strategies in the context of an epidemic are needed to feed into decision-making models for complex supply chains of high-demand essential products, in particular.

### Theoretical implications

With this study, we propose a research agenda for upcoming scholarship on the topic of COVID-19 challenges in manufacturing. This study is one of the first SLRs available on the topic that investigates how large-scale disruptive events, such as the recent COVID-19 pandemic, can challenge the survival of manufacturing businesses. In the interest of extending holistic insights, we map the challenges with interventions based on operational insights reported by the studies reviewed herein. Additionally, we supplement the interventions proposed in some studies with operational evidence available in other studies to discuss the effectiveness of proposed management interventions. By doing so, we contribute to the existing research by (a) confirming the effectiveness of potential interventions, and (b) questioning the sustainability of those interventions. Such scrutiny of the literature has helped us to identify open research questions and propose a consolidated agenda for ensuring the scholarly progression of COVID-19 challenges in manufacturing by future research. Overall, this SLR complements existing research by thematically synthesizing COVID-19 research to establish an understanding of the pandemic’s impact on manufacturing. These insights will inform the growth of theory and application of management interventions across manufacturing firms.

### Managerial implications

This review bears significant managerial implications. Firstly, it bridges research with practice; by identifying the several managerial actions for the many COVID-19 challenges, we extend a preemptive checklist to provide manufacturing executives and managers with the information needed to prepare for disruptive events. This will help them evaluate how pandemic-ready they are, and assist them in improving their responsiveness to future global emergencies. More specifically, these findings will enable manufacturers to foster a proactive environment, and employ apt response strategies and policies to avert the risks associated with disaster-level events. Such insights are expected to lay the foundation for restructuring supply chains and revisiting production strategies that can together assist manufacturers’ economic recovery from COVID-19 complexities.

Secondly, it is clear from this review that existing manufacturing processes are neither agile nor resilient to disruptive events. Two critical tasks lie ahead for manufacturers: to ascertain the continuity of manufacturing operations, and to attain long-term supply chain resilience to reinforce the continuity of operations under all circumstances (Cai & Luo, 2020; Monostori & Váncza, 2020). This review demonstrates that existing research has unanimously proposed the effectiveness of digital technologies for addressing the aforementioned tasks. In employing such technologies, manufacturing executives and managers have to be prepared to deal with the challenges of bureaucracy, digital upskilling, organizational resistance to change, data ownership, high digital investment costs, data privacy and security, and the dangers of competing with larger tech giants in digitalizing manufacturing processes (Covino et al., 2019; Hallstedt et al., 2020; Liu et al., 2020). In addition, manufacturers need to work together with policymakers, government agencies, educators and corporations to establish long-term objectives and solutions for managing pandemic-level adversities (Wuest et al., 2020).

Thirdly, the study also bears implications for policymakers. The strategies reviewed in this study, specifically the interventions pertaining to government policies, suggest avenues for future policymakers to pursue. Future policy building should in entirety support the growth and sustainability of manufacturing supply chains. Policymakers’ focus should be on building robust networks and agile manufacturing systems (Huang, 2020; Rapaccini et al., 2020) that can sustain disruptions of any scale. The need is for policies and regulatory frameworks to be focused on aspects of financial aids, tax preferences and employment subsidies, resourcing strategies, cooperative manufacturing, manufacturing competitiveness, subsidized digitalization-specific loans, employee training and digital upskilling, protection from liquidity crunches, demand-oriented product and marketing innovations, and entry into international markets and operations across borders.

### Limitations

The first limitation is the limited number of studies on the topic, which is attributed to the fact that this review has been put together during the pandemic. Only 56 publications fit the scope, with many conducted during the early part of the crisis (43 of the 56 papers reviewed here were published in 2020). More remains to be observed, studied, and analysed as the severity of the pandemic in a manufacturing context is yet to be fully captured by research. Limited evidence prevents us from presenting conclusions that encapsulate the full picture. Nevertheless, limited evidence does not mean studies like ours should wait until the full recovery from COVID-19. It is important for research and practice to document and apply learnings as and when they evolve, and although some inferences in this review may be of a tentative or predictive nature, they are still of merit. Future research should build upon this review with supplementary data to validate/dismiss the findings presented herein.

Considering this topic is receiving continuous widespread attention from the academic community given its timeliness, new research papers are flooding the research pool on a daily basis. Our next limitation is pivoted around the planning and scoping process, which was conducted using the Scopus database in the last week of March 2021. Research articles published after this timeline have not been included in this review. As the world fights the pandemic, new research insights are becoming available for manufacturing; future reviews should capture how the holistic impact of COVID-19 on the—economic, environmental, technological and social aspects has since evolved.

Additionally, it is worth noting that out of the 56 publications reviewed here, we found a small proportion of studies (Gu et al., 2020; Hallstedt et al., 2020; Huang, 2020; J. Li, 2020; Luo et al., 2020; Paucar et al., 2020; Payne et al., 2021; Terry et al., 2020) that mention COVID-19 in a manufacturing context without sharing any significant pandemic-related insights. While we do reference these studies to reinforce some of our arguments that indirectly support discussions around challenges and interventions, they do not add any exclusive insights on the topic. For instance, Paucar et al. (2020) discuss delayed production processes, and reference COVID-19 to suggest that lean manufacturing can accommodate social distancing.

## Conclusions

It has been nearly 16 months since the onset of the COVID-19 pandemic, and there is, as yet insufficient evidence to be certain how the emerging challenges will evolve in manufacturing, or how they could be successfully managed. Therefore, with this review, we collate the challenges documented since the pandemic’s onset, alongside management interventions, by undertaking a SLR. In consolidating the research on the current state and future of the pandemic-afflicted manufacturing industry, we come across an array of challenges—related to lockdowns, product shortages, safety protocols, workforce shortages, and other financial burdens. Existing research is extremely critical in highlighting manufacturers’ mistakes resulting in frail and fractured supply chains, but it also recognizes their efforts in pursuing potential avenues to combat COVID-19 challenges. In managing these challenges, several interventions, including the use of digital technologies, repurposing, localizing, service provision, flexible manufacturing, coopetition, and targeted government policies have been identified. This review demonstrates that, with the right tools and interventions, the manufacturing industry will eventually bounce back as a reinforcing pillar of the world economies. All of these interventions hold merit, but some need further validation, and so we put together a robust and comprehensive research agenda that structures them as avenues for future research. It is important that future research is not restricted to investigating the existing challenges of COVID-19; the scope instead should be broader—one that focuses on resilient manufacturing, capable of absorbing and minimizing the impact of any wide-scale disruption.
